# mTOR pathway activation in multiple myeloma cell lines and primary tumour cells: pomalidomide enhances cytoplasmic-nuclear shuttling of mTOR protein

**DOI:** 10.18632/oncoscience.148

**Published:** 2015-04-06

**Authors:** Tommasina Guglielmelli, Emilia Giugliano, Vanessa Brunetto, Ida Rapa, Susanna Cappia, Jessica Giorcelli, Sokol Rrodhe, Mauro Papotti, Giuseppe Saglio

**Affiliations:** ^1^ Department of Clinical and Biological Sciences, University of Turin and S Luigi Hospital, Orbassano, Turin, Italy; ^2^ Department of Oncology, University of Turin and S Luigi Hospital, Orbassano, Turin, Italy

**Keywords:** multiple myeloma, pomalidomide, mTOR pathway, nuclear, AKT

## Abstract

mTOR is a protein kinase that plays a central role in regulating critical cellular processes. We evaluated the activation and cellular localization of the mTOR pathway in multiple myeloma (MM) and analyzed the role of pomalidomide in regulating mTOR. By immunohistochemistry cytoplasmic p-mTOR stained positive in 57 out 101 (57.6%) cases with a nuclear p-mTOR localization in 14 out 101 samples (13.8%). In the 70 MM samples analyzed for the entire pathway, p-mTOR expression significantly correlated with p-AKT, p-P70S6K, and p-4E-BP1 suggesting that the AKT/mTOR pathway is activated in a subset of MM patients. Immunofluorescence assays demonstrated that mTOR protein is distributed throughout the cytoplasm and the nucleus at baseline in MM cell lines and in plasma cells of 13 MM patients and that pomalidomide facilitated the shift of the mTOR protein in the nucleus. By western blotting, treatment with pomalidomide increased nuclear mTOR and p-mTOR expression levels in the nucleus with a concomitant decrease of the cytoplasmic fractions while does not seem to affect significantly AKT phosphorylation status. In MM cells the anti-myeloma activity of pomalidomide may be mediated by the regulation of the mTOR pathway.

## INTRODUCTION

Multiple myeloma (MM) is a malignant plasma cell disorder and remains an incurable disease. The availability of agents such as the proteasome inhibitor bortezomib and the immunomodulatory agents lenalidomide and pomalidomide has considerably increased the treatment options of MM patients [1, 2]. New drugs and new combinations are needed to further improve MM patients' survival. Preclinical data show that mammalian target of rapamycin (mTOR) inhibitors such as rapamycin, temsirolimus and everolimus, may be potential target therapies for MM patients, especially if associated with other drugs [3-5]. mTOR is a serine-threonine protein kinase that belongs to the phosphoinositide 3-kinase (PI3K)-related kinase family. It plays a central role in regulating critical cellular processes such as growth, proliferation, cytoskeletal organization, transcription, protein synthesis and ribosomal biogenesis [6, 7]. In mammals, there are two mTOR protein complexes: the regulatory associated protein of mTOR (Raptor)-G protein β-subunit-like protein (GβL)-mTOR complex (mTORC1) and the rapamycin-insensitive companion of mTOR (Rictor)-GβL-mTOR complex (mTORC2). mTORC1 directly phosphorylates the two key translation regulators p70 ribosomal S6 kinase (P70S6K) and eukaryotic initiation factor 4E binding protein 1 (4E-BP1). Phosphorylation of P70S6K stimulates ribosome biogenesis while multi-site phosphorylation of 4E-BP1 results in its dissociation from elF4E thereby allowing elF4E to enhanced cap-dependent translation of oncogenic cap-mRNAs such as MYC, HIF-1 and Cyclin D1 [8-10]. Previous studies demonstrates hyper activation of mTOR in myeloma downstream of PI3-K/AKT [11, 12]. Moreover the PI3K/AKT/mTOR pathway is one of the major pathways mediating cytokine-induced MM cell proliferation, survival and development of drug resistance [12, 13]. The Rictor-GβL-mTOR complex (mTORC2) is relatively rapamycin-insensitive and has a key role in cell survival and proliferation through the AKT phosphorylation occurring on serine 473 [14]. The AKT S473 phosphorylation is relatively specific for MM tumour cells, as adjacent non-malignant hematopoietic cells in patient marrows are usually negative for S473 staining. MM cell line studies suggest that an additional TORC2 target, SGK (glucocorticoid-inducible kinase), is also up-regulated, as its substrate, NDRG1 (N-myc down regulated gene 1), is hyper phosphorylated. Moreover TORC2 is currently known to directly regulate protein kinase C-α (PKC-α) [6, 15].

Consistent with its primary target being the translation machinery, mTOR is predominantly localized in the cytoplasm, associated with a variety of intracellular membrane structures [16, 17]. However, a nuclear localization of mTOR has been initially found in rhabdomyosarcomas (Rh30 and Rh41), human fibroblasts (IMR90) and HCT8 colon carcinoma cells [18]. Furthermore, mTOR becomes nuclear in HEK293 cells treated with leptomycin B, a specific inhibitor of nuclear export receptor Crm1, suggesting that mTOR is a cytoplasmic-nuclear shuttling protein [19]. Pomalidomide is a novel IMID® immunomodulatory drug approved for the treatment of MM by FDA in February 2013. Immunomodulatory drugs (thalidomide, lenalidomide and pomalidomide) possess several activities: immunomodulatory (by enhancing expansion of NKT cells and by reducing regulatory T cell–Tregs-); antiangiogenetic (by decreasing the expression of VEGF and bFGF, thereby inhibiting new blood vessel formation); anti-inflammatory (by inhibiting the production of TNFα); anti-proliferative effects (by inhibiting the activity of NF-kB, C/EBPβ and CDKs) [20]. Cereblon (CRBN) has been considered one of the immunomodulatory drugs target proteins: CRBN expression is required for lenalidomide activity and its deregulation confers resistance to drug treatment [21]. *In vitro* studies showed that pomalidomide is 10-fold more potent than lenalidomide in inhibiting TNFα; pomalidomide has distinct mechanisms of action compared with lenalidomide including direct anti-proliferative (by up-regulation the expression of p21 WAF1 tumor suppressor gene) and pro-apoptotic effects (by enhancing MM sensitivity to Fas-induced and TRAIL/Apo2L-induced apoptosis via a caspase-8-dependent mechanism) [22]. A recent phase 1 trial suggests the potential of lenalidomide-everolimus combination therapy in relapsed/refractory MM patients [23]. This combination is based on preclinical studies showing synergistic activity of mTOR inhibitors with lenalidomide and their ability to overcome the protective effects of growth factors in the myeloma tumour milieu [4]. The molecular mechanism by which these drugs interfere seems to include the mitogen-activated protein kinase (MAPK) and the PI3K/AKT kinase pathways but is not known completely.

The aim of this work is to study the activation of the AKT/mTOR/P70S6K/4E-BP1 pathway and its prognostic impact in MM patients. We also evaluate cellular localization of mTOR protein in MM cell lines and in primary tumour cells. Moreover the role of the pomalidomide in regulating the mTOR pathway is analysed.

## RESULTS

### Effect of pomalidomide on tumour cell proliferation and apoptosis

**Figure 1:**
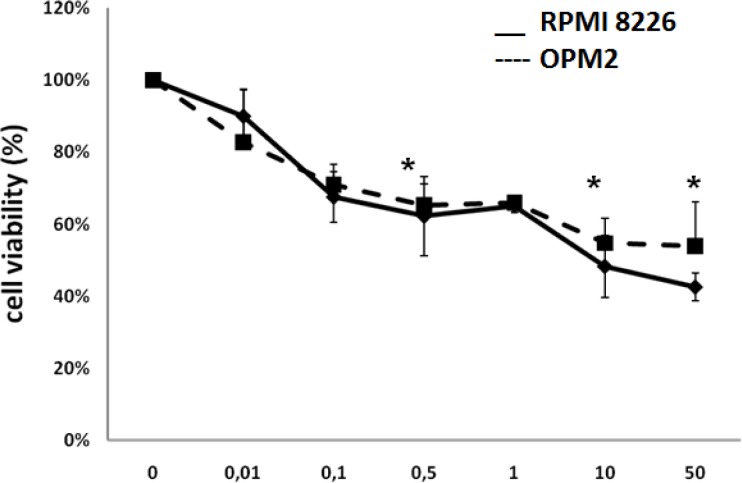
Pomalidomide reduces the viability of MM cell lines Cells were cultured with concentration of pomalidomide ranging from 0.01 μM to 50 μM. Pomalidomide significantly suppressed proliferation of RPMI8226 and OPM2 cells at 48 h with IC50 values of 8 μM and 10 μM. Data are presented as mean +/− SD.*P<0.05.

OPM2 and RPMI8226 cell lines were cultured at 24h and 48h and incubated with increasing doses of pomalidomide (ranging from 0.01 μM to 50 μM). MTT assay demonstrates that pomalidomide significantly reduced cell viability of RPMI8226 and OPM2 cells at 48h with IC50 values of 8 μM and 10 μM, respectively (FIG 1).

**Figure 2:**
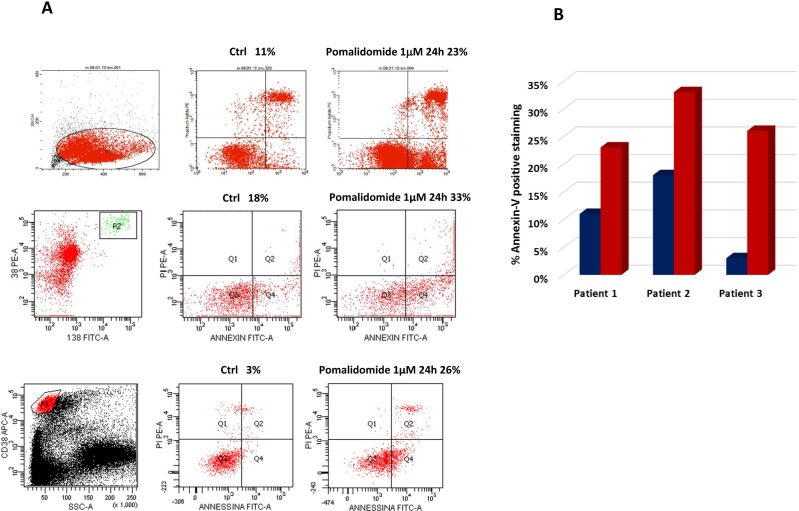
Anti-myeloma activity of pomalidomide on CD138+ cells from 3 MM patients CD138+ cells were selected and apoptosis with pomalidomide 1 μM for 24 h was evaluated with flow cytometry measurements. Annexin V+ /PI- cells were considered in early apoptosis phase. (A). Blue columns represent controls (11%,18%,3%); red columns represent % apoptosis after treatment with 23%, 33% and 26% annexin- V+ /PI-e cells (B). Data are presented as mean +/− SD. *P<0.05.

The apoptotic effect of pomalidomide was evaluated on MM cell lines and patients' MM cells by flow cytometry. MM cell lines were incubated with Pomalidomide 0.01, 0.1, 1, 10 and 50 μM at 24h, 48h and 72h. Plasmacells were labelled with annexin V conjugated with fluorescein isothiocyanate and propidium iodide and annexin V+ /PI-cells were considered in early apoptosis phase. No significant apoptosis was detected in RPMI8226 and OPM2 cells (data not shown). Plasmacells from three MM patients were identified using anti-CD38 antibody and incubated with pomalidomide 1 μM for 24h: pomalidomide significantly induced apoptosis cell death (23%, 33% and 26% versus controls 11%,18%,3%, P<0.05) (FIG 2).

### Localization of mTOR protein by confocal microscopy

**Figure 3:**
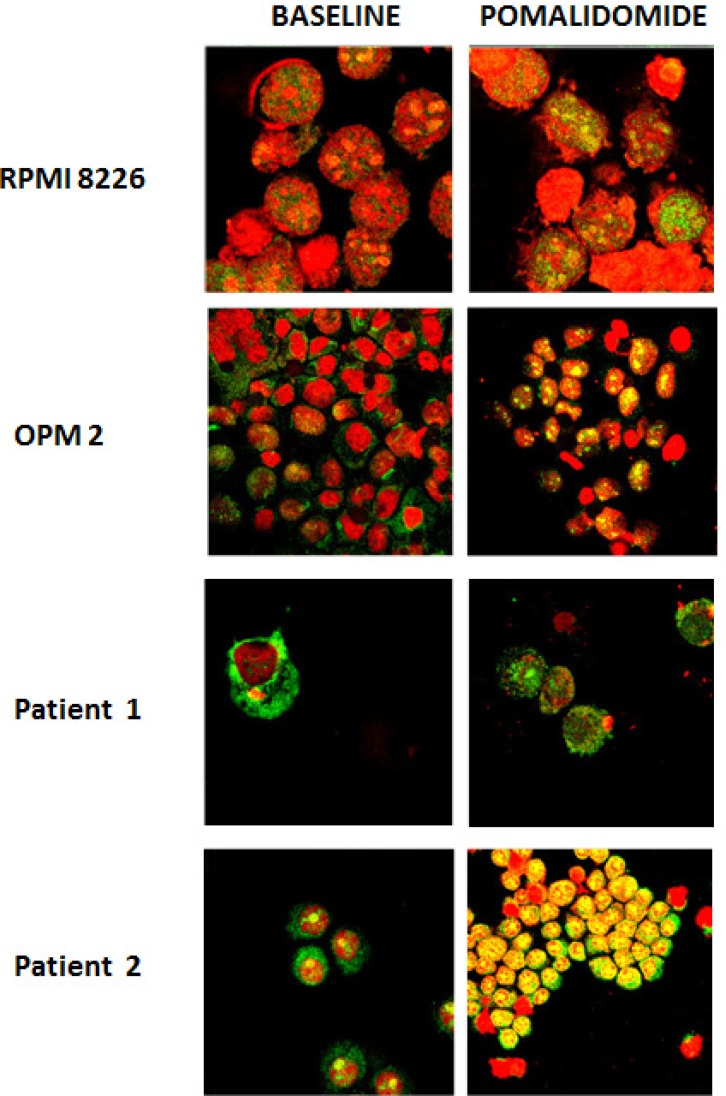
Immunofluorescent images of mTOR protein localization in RPMI8226 and OPM2 cells and primary myeloma cells The mTOR protein is distributed throughout the cell cytoplasm and nucleus at baseline in RPMI8226 and OPM2 cells. After incubation with pomalidomide 10 μM for 48 h, both cell lines demonstrated an increase of the nuclear mTOR protein. CD138 + cells from patient 1 evidences cytoplasmic localization of the mTOR protein in basal condition and the appearance of the nuclear localization after pomalidomide treatment. CD138 + cells from patient 2 has a nuclear mTOR localization in basal condition and an increase of the protein after drug incubation. Red: nuclear Dna (propidium ioduro), green: mTOR antibody, yellow: co-localization signal. Scale bar = 5 μm.

**Figure 4:**
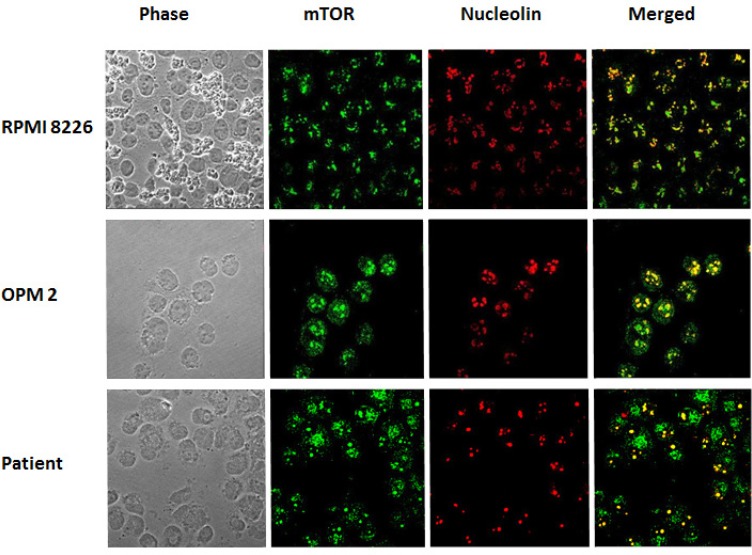
Co-localization of mTOR antibody and nucleolin in RPMI8226, OPM2 cell lines and in CD138 + cells mTOR antibody stains many cell nuclei and cytoplasm (green). Antibody to nucleolin stains nucleoli and many dot-like speckles (red). Nuclei which stain for mTOR (green) merge with nucleolin (red) to show co-localization (yellow). The merge occurs both in nucleoli (which are phase dense) and in some nuclear speckles. Scale bar = 10 μm.

Immunofluorescence assays using antibodies against mTOR protein were performed on RPMI8226 and OPM2 cell lines and on CD138 positive cells from thirteen MM patients. We evidenced that in RPMI8226 and OPM2 cells, the mTOR protein is distributed throughout the cell cytoplasm and nucleus at baseline. After incubation with pomalidomide 10 μM for 48 h, MM cell lines demonstrated an increase of the nuclear mTOR protein (FIG 3). CD138+ cells from four multiple myeloma patients were analyzed at baseline and after pomalidomide treatment 1 μM for 24 h. Nuclear mTOR localization was detected in three out four cases at baseline. An increase of the nuclear mTOR protein after pomalidomide treatment was detected in three patients: two of them had a nuclear mTOR localization at baseline while the remaining patient acquired nuclear mTOR localization after pomalidomide treatment (FIG 3). We compared mTOR and nucleolin co-localization in RPMI8226 and OPM2 cells and in CD138 positive cells from nine MM patients. MM cells exhibit varying staining patterns with the mTOR antibody: the nuclear patterns included punctate bodies, small dot-like speckles and speckles. On the same cells, the nucleolin antibody stained nucleoli and some dot-like speckles. The co-localization of mTOR protein (green) and nucleolin (red) occurred frequently in nucleoli (which are phase dense in FIG 4) and in some nuclear speckles, and exhibited merged (yellow) regions.

### Immunohistochemistry

**Figure 5:**
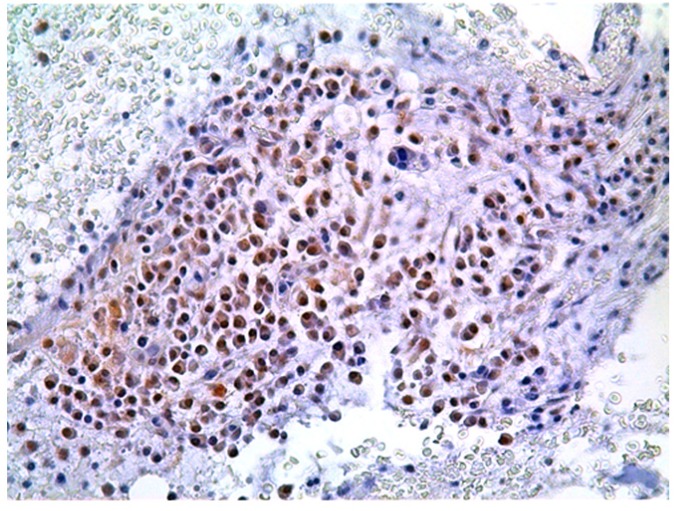
Representative immunohistochemical staining of the mTOR protein nuclear localization in MM bone marrow sample Original magnification, 200x.

P-mTOR staining was analyzed on bone marrow sections from 101 MM patients. Globally, 57 out 101 (56.4%) cases demonstrated p-mTOR positivity with a predominant cytoplasmic staining pattern. A nuclear p-mTOR staining was also demonstrated in 14 out 101 cases analyzed (13.9%) (FIG 5). All but one patient showed both nuclear and cytoplasmic p-mTOR staining. Immunohistochemistry for p-AKT, p-P706SK and p-4E-BP1 were also performed on 70 MM cases on the basis of the sample availability. The median cut-off value was chosen to identify those patients who stained positive for each antibody. Specimens with an HSCORE of ≥30 and ≥45 were classified as p-mTOR (range 0-285) and p-AKT (range 0-285) positive while for p-P70S6K and p-4E-BP1 specimens were considered positive when the HSCORE was ≥60 (range 0-285) and ≥40 (0-270), respectively. Globally, 44 out 70 (62.8%) MM patients stained positive for p-mTOR and 37 (52.8%), 42 (60%) and 38 (54.3%) cases stained positive for p–AKT, p-P706SK and p-4E-BP1, respectively.

**Figure 6:**
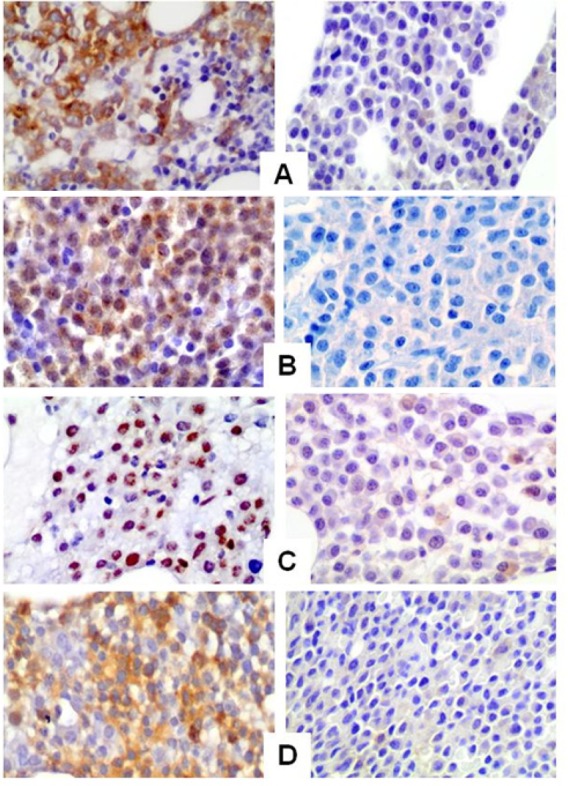
Representative immunohistochemical staining of MM bone marrow samples for p-AKT (A), p-mTOR (B), p-P706SK (C) and p-4E-BP1 (D). The right panel shows negative immunoreactivity, while the left panel shows strong positive immunoreactivity for the specific antibodies Original magnification 200x.

P-mTOR and p-AKT are expressed in MM patients with a predominance of cytoplasmic staining pattern; p-P70S6K and p-4E-BP1 are also detected with nuclear staining pattern (FIG 6).

Overall, p-mTOR expression significantly correlates with p-AKT (concordance r=0.29, P=0.05), p-P70S6K (r=0.35, P=0.001), and p-4E-BP1 (r=0.41, P=0.0001) positive staining consistent with the hypothesis that the mTOR pathway is activated in a subset of MM patients.

Clinical data were available on 75 MM patients (Table 1) (57 newly diagnosed and 18 relapsed disease) as follow: median age 66 years (range 38-87); monoclonal component IgG 42 cases (56%), IgA 20 cases (26.7%), 12 light chain myelomas (16%) and 1 non-secreting myeloma (1.3%); LDH serum levels higher than normal, 13 patients (17.3%); serum levels of β2-microglobulin higher than 3.5 mg/dL and 5.5 mg/dL 24 (32%) and 17 (22.6%) cases, respectively; Hb <10 gr/dL 24 patients (32%); osteolytic lesions 45 cases (60%); ISS stage I, II, III in 31 (41.4%), 27 (36%) e 17 (22.6%) patients, respectively.

**Table 1:** Clinicopathological characteristic of 75 MM patients and relation with p-mTOR expression

Variable	N=75	p-mTOR positive staining	p value
Age, years			
>65	43	26/43	n.s
≤65	32	16/32	
M component,			
IgG	42	23/42	n.s
IgA	20	20-Dec	
Light chain	12	12-Jul	
Non secreting	1		
β2-microglobulin, mg/dL			
≤3.5	34	28/34	n.s.
>3.5	24	14/24	
>5.5	17	17-Sep	
LDH serum level, U/L			
≤243	62	30/62	0.004
>243	13	13-Dec	
Hb serum level, gr/dL			
≥ 10 g	51	29/51	n.s
< 10	24	13/24	
Osteolitic lesions			
Presence	45	24/45	n.s
Absence	30	18/30	
ISS stage			
I	31	17/31	n.s
II	27	16/27	
III	17	17-Sep	
Disease phase			
Diagnosis	57	33/57	n.s
Relapse	18	18-Nov	

### Statistical correlation between p-mTOR expression and clinical variables clinical data

Clinical data were available on 75 MM patients (57 newly diagnosed and 18 relapsed disease) as follow: median age 66 years (range 38-87); monoclonal component IgG 42 cases (56%), IgA 20 cases (26.7%), 12 light chain myelomas (16%) and 1 non-secreting myeloma (1.3%); LDH serum levels higher than normal, 13 patients (17.3%); serum levels of β2-microglobulin higher than 3.5 mg/dL and 5.5 mg/dL 24 (32%) and 17 (22.6%) cases, respectively; Hb <10 gr/dL 24 patients (32%); osteolytic lesions 45 cases (60%); ISS stage I, II, III in 31 (41.4%), 27 (36%) e 17 (22.6%) patients, respectively. Cytoplasmic p-mTOR positive staining (HSCORE ≥30) correlates with high LDH serum levels (P=0.004, chi-square 8.4). These data suggest that the expression of m-TOR protein in MM patients may have clinical and prognostic implications.

### Western blotting

**Figure 7:**
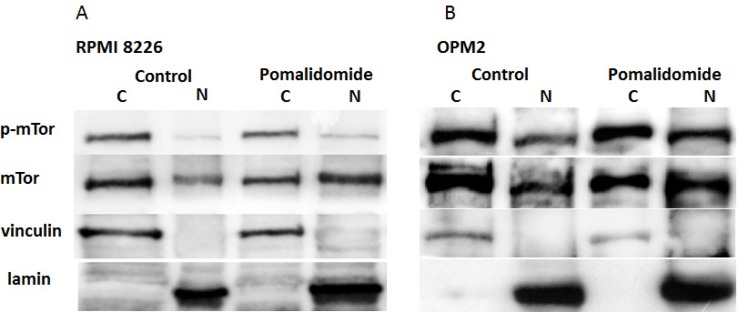
Pomalidomide strengthens cytoplasmic-nuclear shuttling of mTOR and p-mTOR protein in RPMI8226 (A) and OPM2 (B) cells Cellular distribution of mTOR and p-mTOR was determined in cytoplasmic and nuclear fractions at baseline (control) and with pomalidomide treatment 10 μM at 48 h by western blotting in RPMI8226 (A) and OPM2 (B) cells.

**Figure 8:**
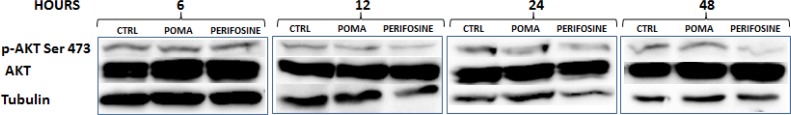
Pomalidomide does not affect AKT and p-AKT S473 levels in OPM 2 cells Cells were cultured with pomalidomide 10 μM and perifosine at 20 μM at 6, 12, 24 and 48 h. Perifosine, as expected, reduces p-AKT levels at 12 h.

Cytoplasmic and nuclear distribution of mTOR and p-mTOR was investigated by western blot in OPM2 and RPMI8226 cells. Cytoplasmic and nuclear fractions were obtained following specific protocol described below. Western blot analysis revealed that mTOR and p-mTOR can be detected in the cytoplasm and in the nucleus at baseline in both myeloma cell lines. As expected, the mTOR and p-mTOR protein levels were significantly higher in the cytoplasm when compared to the nucleus. Treatment with pomalidomide 10 μM for 48h, increased nuclear mTOR and p-mTOR protein levels in the nucleus with a concomitant reduction of the cytoplasmic mTOR fraction in RPMI8226 and OPM2 cells. The p-mTOR cytoplasmic fraction was reduced in RPMI8226 but was variable in OPM2 cells (Figure 7 A,B). Pomalidomide 10 μM did not affect AKT and p-AKT S473 protein levels while perifosine (20 μM), as expected, reduced p-AKT S473 levels in OPM-2 cells (Figure 8). RPMI 8226 did not demonstrate constitutive activation of p-AKT (data not shown).

## DISCUSSION

In this study, we found cytoplasmic p-mTOR expression in almost half of the multiple myeloma samples investigated by immunohistochemistry. Moreover, p-mTOR expression was significantly correlated with the expression of p-AKT, p-P70S6K, and p-4E-BP1, suggesting an activation of the mTOR pathway in a subset of MM patients.

Early studies identified activation of the P13K/AKT pathway in MM cell lines and in primary MM cells [24-26]. Shi et al [11, 12] demonstrated that IL-6 or IGF-1 exposure up-regulated phosphorylation of p70S6K and 4EBP-1 and that the mTOR inhibitors may prevent activation of the mTOR pathway by inhibiting cytokine-dependent myeloma cell growth. In addition to the stimulating effects of IL-6 and IGF-1, loss of function mutations of the tumour suppression gene PTEN in several MM cell lines, results in phosphorylation of the mTOR substrates [12]. More recent data suggest that in MM, RAS mutations could activate TORC1 and TORC2 complexes and this events was correlated with an aggressive phenotype [15, 27]. Our study suggests that in MM specimens mTOR activation correlates with AKT activation, and thus usually occurs downstream of PI3-K/AKT.

Activation of the mTOR signalling pathway has recently been found to be strongly implicated in several human cancers and in age-related disease [28]. In early stage, triple negative (estrogen and progesteron receptor, Her2 amplification) breast carcinomas, phosphorylated mTOR expression significantly correlated with worse overall survival and recurrence-free survival [29]. In oesophageal squamous cell carcinoma, high levels of phosphorylated mTOR were significantly associated with shortened disease specific survival and mTOR expression remained an independent adverse prognostic factor in multivariate analysis [30]. Similar data were obtained in early stage Non Small Cell Lung Cancer where angioinvasion and mTOR expression were significant predictors of poor survival at both univariate and multivariate analysis [31]. The relevance of mTOR signalling in Renal Cell Carcinoma (RCC) is highlighted by the success in using mTOR inhibitors (temsirolimus and everolimus) to treat patients with advanced disease [32]. A recent work suggests that in clear RCC a cumulative number of altered biomarkers in mTOR pathway (p-AKT, p-P706SK, p-mTOR, HIF-1alfa, Raptor, PTEN, P13K, p-4EBP-1) correlates with aggressive tumour biology (tumour stage and grade) and inferior disease outcome [33]. Our study also demonstrated that phosphorylated mTOR expression, as detected by immunohistochemistry, was significantly correlated with high LDH serum levels. Recent data highlighted the importance of this variable in high risk myeloma. A new definition of this latter category is ongoing and it includes, in addition to unfavourable cytogenetic profile [t(4;14) and/or del(17p)], International Staging System 3 (β2microglobulin >5.5 gr/dL) and high LDH serum levels [34].

Activation of the PI3K/AKT/mTOR pathway is frequently implicated in resistance to anticancer therapies, including tyrosine kinase inhibitors, radiation, and cytotoxic drugs. Moreover preclinical evidence shows that inhibitors of PI3K or mTOR can restore sensitivity in breast cancer, non-small-cell lung cancer and glioblastoma cells resistant to biologic and cytotoxic drugs [35]. The possibility that p-mTOR expression may confer adverse prognosis in MM patients by activating its own pathway or by conferring resistance to drugs should be investigated.

mTOR protein localization is mainly cytoplasmic. However nuclear localization has been found in several tumors [36-38]. Here we found that in multiple myeloma cell lines and in primary myeloma cells mTOR is distributed throughout the cell cytoplasm and also nucleus at baseline. By immunofluorescence and confocal microscopy, cytoplasmic and nuclear distribution of mTOR was detected in RPMI8226 and OPM2 cell lines and in plasma cells from 12 out 13 MM patients, as also confirmed by co-localization experiments with the nuclear protein nucleolin. A nuclear p-mTOR staining was also demonstrated in 14 out 101 cases (13.9%) by immunohistochemistry. Results from western blot analysis on RPMI8226 and OPM2 cells revealed that mTOR and p-mTOR can be detected in both the cytoplasm and the nucleus. This is the first report of a mTOR nuclear localization in multiple myeloma. The nuclear distribution of mTOR is surprising and intriguing, as this pathway regulates cytoplasmic targets. In Saccharomyces cerevisiae the mTOR kinase localizes to tRNA and 5S rRNA genes and, by interacting with the transcriptional factor TFIIIc, recognizes the promoter of this genes. Moreover mTOR can phosphorylates Maf1, a repressor that binds and inhibits RNA pol III, therefore enhancing its inhibitor function [39]. In mammals, a previous work suggests that in HEK 293 cells mTOR binds to the promoters of RNA polymerase I- and III-transcribed genes and that this association is regulated by growth signals and is sensitive to rapamycin [40]. In our work, in MM cells, mTOR was mainly localized in the nucleolus. As its primary function is to transcribe and modify rRNA and to assembly ribosomes, this localization may suggest a role in regulating such functions.

Treatment with pomalidomide performed by western blot, also increased nuclear mTOR protein levels in RPMI8226 and OPM2 cells with a concomitant reduction of the cytoplasmic fraction. We also observed a cytoplasmic-nuclear shuttling of the p-mTOR protein with a cytoplasmic fraction reduction in RPMI8226 cells and a variable amount in OPM2 cells. This work adds more information about pomalidomide mechanisms of action in multiple myeloma. A previous study analysed cytoplasmic-nuclear shuttling of mTOR protein in both human embryonic kidney (HEK) 293 cells and monkey kidney epithelial CV-1 cells [19]. By using leptomicycin B, a specific inhibitor of nuclear export receptor Crm1, authors demonstrated that mTOR is a cytoplasmic-nuclear shuttling protein. Moreover, inhibition of mTOR nuclear export by leptomicin and the addition of exogenous nuclear import signals to mTOR, coincides with diminished p70S6K activation and 4E-BP1 phosphorylation. It should be possible that nuclear shuttling of the mTOR protein in MM cells, as induced by pomalidomide, may regulate the mTOR pathway both by diminished the cytoplasmic p-mTOR fraction and by direct regulation of the protein synthesis machinery via extracellular signals.

Previous studies on the anti-proliferative effects of these compounds suggested that they may inhibit the activity of PI3K/AKT pathway. Lenalidomide is able to inhibit the phosphorylation of AKT in response to growth factors in endothelial cells that correlates with anti-migratory and anti-angiogenic effect [41]. Furthermore lenalidomide significantly attenuates VEGF-induced AKT phosphorylation in endothelial cells thus interfering with endothelial cell survival, migration and vessel formation [42]. In our work pomalidomide does not affect directly AKT S473 phosphorylation status in OPM 2 cells suggesting that the drug is not active on mTORC 2. Recent data highlight that phosphorylation of AKT at S473 by mTORC2 is necessary for full AKT activation [43] so we can suppose that pomalidomide activity on mTOR and p-mTOR proteins may occur independently from AKT.

More studies are needed to evaluate the significance of nuclear mTOR localization, transport and function in MM. Our work suggests a way to identify a subgroup of MM patients with mTOR pathway activation in order to select patients that may benefit from a target therapy. Moreover, a better knowledge about pomalidomide mechanisms of action may explain its efficacy in the treatment of MM patient when used either alone or in combination with other drugs.

Pomalidomide activity on the mTOR activation status may also suggest pomalidomide-mTOR inhibitor combinations in order to enhance drugs activity and efficacy in MM and solid tumors too.

## MATERIALS AND METHODS

### Cell lines

RPMI8226 were kindly provided by Dr Roberto Piva (Department of Pathology and CeRMS, University of Turin). OPM2 was purchased from DSMZ (Braunschweig, Germany). All MM cell lines were cultured in IMDM (Sigma-Aldrich Corp. St. Luis, MO, USA) containing 10% fetal bovine serum, 2mM L-glutamine (Gibco-Invitrogen, Grand Island, NY), 100 U/mL penicillin, and 100 μg/mL streptomycin (Gibco).

### Drugs

Pomalidomide was obtained from Celgene. The drugs were dissolved in dimethylsulfoxide (DMSO; Sigma) at a concentration of 200 mM and were stored at −20°C until use. Pomalidomide was diluted in culture medium (0,01-50 μM) with less 0,1% DMSO immediately before use. Perifosine was supplied by Selleckchem, dissolved in DMSO <1mg/ml and stored at −80°C until use.

### MTT colorimetric survival assay

OPM2 and RPMI8226 cell lines were plated in 96-wells plates for 24h and then treated with increasing concentrations of pomalidomide (ranging from 0.01 μM to 50 μM) for 24 h and 48 h, respectively. A 3-(4,5-dimethylthiazol-2-yl)-2,5-diphenyl tetrazolium bromide (MTT, Sigma-Aldrich Corp. St. Luis, MO, USA) assay was performed according to the manufacturer's instructions. Assorbance was measured at 570 nm (with correction using readings at 690 nm) using a microplate reader (Asys UVM 340). The cytotoxicity was expressed as the percentage of cells surviving relative to untreated cultures. The half maximal inhibitory concentration (IC50 values) was calculated using the ED50 Plus v1.0 online software. All experiments were repeated at last 3 times, and each experimental condition was performed in triplicate. Data are presented as mean ±SD.

### Apoptosis assay

Apoptosis was evaluated by flow cytometry for the detection of annexin V-positive cells. MM cell lines were incubated for 24 h, 48 h and 72 h with media alone or with varying concentration of pomalidomide (0.01, 0.1, 1, 10 and 50 μM).

Bone marrow aspirates were subjected to Ficoll Hypaque gradient centrifugation and mononuclear cells were suspended in IMDM media containing 20% fetal bovine serum, 2 mM L-glutamine, 100 U/mL penicillin, and 100 μg/mL streptomycin. Cells were placed at in a plate at a concentration of 2 × 106 cells/mL; pomalidomide was added to the medium at the concentration 1 μM. Patient plasma cells were cultured for 24 h and then harvested. Plasma cells were identified by using anti-CD38 antibody (Becton Dickinson, San Jose Ca). Apoptosis was quantified using Annexin V-FITC/Propidium iodide apoptosis detection kit, as per manufacturer's instructions (Immunostep, Salamanca, Spain), followed by an analysis on FACS Canto II, (Becton Dickinson, San Jose Ca).

### Confocal Microscopy

RPMI8226 and OPM2 cells were incubated with 10 μM of pomalidomide for 48 h. Bone marrow samples of MM patients were collected during standard procedure after informed consent and subjected to Ficoll-Hypaque gradient centrifugation (Biochrom, Berlin, Germany). The plasma cell populations were positively selected by the immunomagnetic method using CD138 microbeads (Miltenyi Biotec, Bergisch Gladbach, Germany). Briefly, 10 μL of CD138 microbeads and 90 μL of buffer M (PBS, pH 7.2, bovine serum albumin 0.5%, ethylenediaminetetraacedic acid 2 mmol/L) for 5×106 mononuclear cells were added and incubated for 15 minutes at 4°C. After washing, the cells were layered on a VarioMACS separation column (Miltenyi Biotec) following the manufacturer's instructions. CD138 positive cells were removed from the column, and their purity (90%) was assessed by cell morphology. Plasma cells were incubated with 1 μM of pomalidomide for 24 h.

Slides from MM cell lines and primary MM cells were subjected to immunofluorescence with mTOR antibodies (Abcam, Cambridge, UK) at 1:100 dilution for 2 h at room temperature and washed extensively in PBS. Antibody–antigen complexes were detected by incubation for 60 min with a goat anti-rabbit Alexa Fluor 488-conjugated secondary antibody (Jackson Immuno Research Laboratories, Baltimora Pike West Grove PA). Slides were washed in PBS and treated with propidium iodide (Sigma-Aldrich Corp. St. Luis, MO, USA) for 5 min to stain the nucleus. Cells were analyzed with a Confocal and Fluorescence Microscopy Lab (Zeiss LSM 510; Axiovert 200 with cd camera and computer-driven image acquisition; semi-automated Eppendorf micro-manipulator) equipped with a 40X Plan Neofluar objective. To confirm the nuclear localization of mTor we perform a Z stack of 9-20 slice with an interval of 0.5 μm. For nucleolin staining, C23 (H-6), a mouse monoclonal antibody was used at 1:100 dilution (Santa Cruz Biotechnology, California) and the antibody-antigene complexes were detected by incubation for 60 min with a goat anti-mouse Alexa Flour 568-conjuated secondary antibody.

### Immunohistochemistry and patients

Immunohistochemical studies was conducted on bone marrow biopsies of 101 symptomatic MM patients fulfilling the International Myeloma Working Group Diagnostic Criteria [44]. The selection was based on the availability of bone marrow biopsies plus clinical data. Bone marrow aspirates and biopsies were taken from each patient at the same time during standard diagnostic procedure after informed consent. Clinical data of MM patients are summarized in Table 1. Serial section to that stained with hematoxylin and eosin were collected onto charged slides for immunohistochemistry. An automated immunoperoxidase procedure was employed (Autostainer, DakoCytomation, Glostrup, Denmark) using standard detection methods based on biotin-free reagents (peroxidase conjugated dextran polymers; EnVisionTM + System/HRP,Rabbit/Mouse, DakoCytomation). Before starting the immune reaction, endogenous peroxidase activity was blocked with hydrogen peroxide, and antigen retrieval was obtained by microwaving (0.1 M citrate buffer, pH 6.0) for all antibodies tested. Diaminobenzidine was used as chromogen to reveal immune reaction.

Neoplastic plasma cells were counted on slides immunostained with CD138 antibody (clone MI 15, diluition 1:150; Dako Cytomation), rabbit polyclonal Kappa (diluition 1:50000, Ylem, Rome, Italy) and rabbit polyclonal Lambda (diluition1: 50000, Ylem).

The primary antibodies tested were rabbit monoclonal anti p-mTOR (Ser2448) (clone 49F9, diluition 1:50; Cell Signaling Technology, Beverly, MA), rabbit monoclonal anti p-AKT (Ser473) (clone 736E11, diluition 1:40; Cell Signaling), rabbit monoclonal anti p-4E-BP1(Thr37/46) (clone 236B4, diluition1:350; Cell Signaling), mouse monoclonal anti p-P70S6-Kinase (Thr389) (clone 1A5, diluition 1:300; Cell Signaling).

The slides were counterstained with Mayer hematoxylin and evaluated with a Leica DMD 108 Digital Microimaging Device (Leica Microsystems, Milan, Italy).

Slides were scored by two independent observers and all cases were analyzed using a semi-quantitative histologic score method, as described in the literature [45]. Briefly, immunostaining intensity of each case was semi-quantitatively scored as follows: 0, no staining; 1, weak staining; 2, moderate staining; and 3, strong staining. In addition, the percentage of neoplastic cells was evaluated. For each case, a value designated HSCORE was obtained multiplying each intensity by the corresponding percentage of positive cells [HSCORE=Σ(IXPC), where I and PC represent intensity and percentage of cells, respectively].

The expression level of each marker was determined by counting all plasma cells in each bone marrow section. Both the nucleus and the cytoplasm were evaluated for each antibody.

The immunostaining for mTOR pathway molecules were performed using antibodies validated in a previous study on colon cancer [46]. and their specificity was further tested by means of western blot analysis. Negative controls were obtained by omitting the primary antibody on a parallel section of each immunohistochemical run.

### Western Blotting

RPMI8226 and OPM2 cells were incubated with 10 μM of pomalidomide for 48 h and western Blot was performed on nuclear and cytoplasmatic fractions.

Cells were washed twice with ice-cold PBS and pellet was lysed in cytoplasmic extraction buffer containing 10 mM HEPES, pH 7.9, 10 mM KCl, EDTA 0.1 mM, supplemented with protease inhibitors (1 μg/ml aprotinin, 1 μg/ml pepstatin, 1 μg/ml leupeptin) and 1 mM sodium orthovanadate and incubated for 2 min at room temperature and another 10 min on ice.

Disruption of the cell membrane was achieved by the addition of CHAPS (3-[(3-cholamidopropyl) dimetthylammonio]-1-propanesulfonate) stock solution (10x), at final concentration of 0.6% [47].

Gently mix and broken cells are then homogenized by passing them through a 19-g needle for three times. Cytoplasmic extract was separated by centrifugation at 4°C and 14000 RPMI for 5 min and was stored at −80°C. The remaining pellet contains nuclei was washed three times in extraction buffer supplemented with 0.6% CHAPS and then centrifugated. The nucleic pellet was lysed in nuclear extraction buffer (20 mM HEPES, pH 7.9, 400 mM KCl, 1 mM EDTA, 1mM EGTA, 10% glycerol, DTT 1 mM, 1 mM sodium orthovanadate), supplemented with protease inhibitors and incubated for 20 min on ice. Supernatant containing soluble nucleic proteins was collected by centrifugation at 4°C and 14000 RPMI for 15 min and stored at −80°C.

Nuclear and cytoplasmic fractions were subjected to sodium dodecyl sulfate-polyacrylamide gel electrophoresis (SDS-PAGE). The separated proteins were transferred to nitrocellulose by elettrophoresis for 1-2 h. Non-specific binding was blocked by incubation with BSA 5% at room temperature for 1 h and membranes were incubated overnight with primary antibody against mTOR and p-mTOR (1:500, Abcam, Cambridge, UK) at 4°C. The membranes were washed four times with TBS-Tween 0,3%, incubated with secondary antibody (1:1000) at room temperature for 1 h, and again washed four times with TBS-Tween 0,3%. The membrane was stripped and reprobed with anti-vinculin, anti-laminin and anti-tubulin antibodies (1:1000)(Santa Cruz Biotechnology, California) to ensure equivalent protein loading. Immunoreactive bands were visualized using Chemidoc TM XRS + with Image Lab TM software molecular imager (Biorad).

Cell lines were treated with Perifosine 20μM for 6 h, 12 h, 24 h and 48 h. Total cell extract was obtained by incubating of cells for 30 min on ice with Ripa Buffer (50 mMTris HCl, 150 mM NaCl, 1% Triton X-100, 10% SDS, 1% Deoxycholic acid, 1 μg/ml aprotinin, 1 μg/ml pepstatin, 1 μg/ml leupeptin and 1 mM sodium orthovanadate). Total extract was separated by centrifugation at 4°C, 13000 RPMI for 10 min andstored at −80°C. Cell lysates were subjected to SDS-page, transferred to nitrocellulose membrane, and immunoblotted with antibodies against AKT and p-AKT (Ser473) (1:1000) and Tubulin (Santa Cruz Biotechnology, California) [48, 49].

### Statistical analysis

Statistical significance of differences observed in drug-treated versus control cultures was determined by using Student t test. The minimal level of significance was considered P <.05. Data was analyzed using GraphPAD Prism software (version 5) and ED50 Plus v1.0 online software.

Fisher exact or chi-square tests were used to test for differences among levels of categoric variables between patients with and without p-mTOR. Spearrman's Rank correlation coefficient was used to evaluate the correlation between p-mTOR, p-AKT, p-P706SK and p-4E-BP1expression. Analysis were performed using the SPSS software package (SPSS, Chicago, IL).
